# Hepatotoxicity Induced by Methyl Eugenol: Insights from Toxicokinetics, Metabolomics, and Gut Microbiota

**DOI:** 10.3390/cimb46100673

**Published:** 2024-10-11

**Authors:** Liang Chen, Jiaxin Li, Qian Li, Qingwen Sun

**Affiliations:** School of Pharmacy, Guizhou University of Traditional Chinese Medicine, Guiyang 550025, China; chenliang029@gzy.edu.cn (L.C.); lijiaxin048@gzy.edu.cn (J.L.); liqian@gzy.edu.cn (Q.L.)

**Keywords:** methyl eugenol, hepatotoxicity, toxicokinetics, metabolomics, gut microbiota

## Abstract

Due to continuous application as a flavoring agent in the pesticide, pharmaceutical, and food industries, methyl eugenol (ME) persists in the environment and causes deleterious impacts including cytotoxicity, genotoxicity, and liver damage. This study utilized a comprehensive approach, integrating toxicokinetics, metabolomics, and gut microbiota analysis, to explore the mechanisms behind ME-induced hepatotoxicity in mice. The study observed significant rises in ALT and AST levels, along with significant weight loss, indicating severe liver damage. Toxicokinetic data showed delayed Tmax and plasma accumulation after 28 days of repeated ME exposure at doses of 20 mg/kg, 40 mg/kg, and 60 mg/kg. The metabolomic analysis pinpointed four critical pathways—TCA cycle; alanine, aspartate, and glutamate metabolism; arginine biosynthesis; and tyrosine metabolism—linked to 20 potential biomarkers. Gut microbiota analysis revealed that extended ME exposure led to microbial imbalance, particularly altering the populations of Akkermansia, Prevotella, and Ruminococcus, which are key to amino acid metabolism and the TCA cycle, thus contributing to hepatotoxicity. However, the causal relationship between changes in gut microbiota and liver metabolite levels still requires further in-depth research. This study underscores the significant role of liver metabolites and gut microbiota in ME-induced liver damage.

## 1. Introduction

Methyl eugenol (ME), a phenylpropanoid found in plants like *Myristica fragrans*, *Rosmarinus officinalis*, and *Acorus tatarinowii*, is a prominent component of essential oils [1]. ME exhibits strong fumigant toxicity and significantly impact both the adult and nymph stages of insects [2]. ME was frequently used as male-specific lure in many trapping programs for certain species of true fruit flies (Diptera: Tephritidae) [3]. Recent research highlights ME′s diverse pharmacological effects, such as reducing inflammation, free radicals, and free macrophages, along with its potent antimicrobial, analgesic, and antiallergic properties, making it a powerful bactericide [4,5]. Its extensive use as a flavoring agent in food, cosmetics, fragrances, and herbal products has drawn considerable interest, especially in the pesticide, pharmaceutical, and food industries [6,7].

However, pervasive daily exposure to ME has raised concerns about its potential ecological and health risks. Despite being classified as Generally Recognized As Safe (GRAS) by the Flavor and Extract Manufacturers Association in 1965, in silico tools have identified possible genotoxic and carcinogenic risks associated with ME [8,9,10]. Experimental studies support these concerns, showing that ME can induce cytotoxicity and genotoxicity in rodents, likely due to sulfotransferase-mediated activation of DNA-reactive electrophilic metabolites [11]. The liver, essential in metabolic processes, is particularly susceptible to these exogenous compounds [12]. A two-year study by the National Toxicology Program found that ME caused neoplastic lesions in the livers of 344 Fischer rats and B6C3F1 mice [13]. The hepatotoxicity and cytotoxicity of ME have been well documented, and metabolic activation has been suggested to be involved in ME-induced toxicities [14]. Following its uptake, ME undergoes Cytochrome P450 and sulfotransferase 1A1-dependent metabolic activation, leading to DNA damage [15]. Additionally, its structural analog, eugenol, has been shown to cause liver damage in mice through glutathione depletion [16]. Despite these findings, the full extent of ME-induced liver injury and the molecular mechanisms underlying its hepatotoxicity remain poorly understood.

ME’s pharmaceutical potential is limited by its poor water solubility and low bioavailability [17]. Since the efficacy and toxicity of compounds are closely tied to in vivo exposure, analyzing the toxicokinetics of ME across different doses is crucial for assessing its toxicity risks and uncovering the mechanisms behind its toxic effects [18].

Metabolomics, a sophisticated branch of omics technology, enables the extensive analysis of metabolic shifts by generating large datasets on internal metabolic processes [19]. By comparing normal and pathological metabolic profiles, this approach helps identify biomarkers and disrupted pathways, offering valuable insights into disease mechanisms [20,21,22]. Its unbiased nature makes metabolomics a valuable tool in toxicology research, where it has been effectively applied in animal studies.

The gut–liver axis, a close anatomical and functional connection between the intestine and liver, plays a pivotal role in metabolizing exogenous substances [23]. The gut microbiota, a diverse microbial community in the gastrointestinal tract, significantly influences this process by either enhancing or counteracting host enzyme activities [24]. Although the role of gut microbiota in drug-induced hepatotoxicity is not fully understood, the existing literature suggests its involvement in toxicokinetics and subsequent toxic responses [25].

In light of these factors, this study aimed to validate the hepatotoxic effects of ME and explore the underlying mechanisms of ME-induced liver toxicity in mice through a comprehensive approach that integrates toxicokinetics, metabolomics, and gut microbiota analysis.

## 2. Materials and Methods

### 2.1. Reagentss and Chemicals

Methyl eugenol (CAS#93-15-2, 98.00% purity) was sourced from Abbott Bio-Technology Co., Ltd. (Chengdu, China). The KOD One™ PCR Master Mix and KOD FX Neo PCR enzyme were supplied by Beijing Bailingke Biotechnology Co., Ltd. (Beijing, China). The Monarch DNA Gel Recovery Kit was obtained from Beijing Hongyue Innovation Technology Co., Ltd. HPLC-grade acetonitrile was acquired from Concord Technology (Tianjin, China). All other chemicals were of analytical grade, and doubly distilled water was utilized throughout the study.

### 2.2. Animal Experiments

Twenty-four male Kunming (KM) mice, aged 6 weeks and specific-pathogen-free (SPF), were sourced from Tianqin Biotechnology Co., Ltd. (Changsha, China). After a one-week acclimatization period, the mice were weighed and randomly allocated into four groups: control, low-dose ME (ME-L), medium-dose ME (ME-M), and high-dose ME (ME-H). The ME-L, ME-M, and ME-H groups received ME dissolved in 0.5% CMC-Na at doses of 20 mg/kg, 40 mg/kg, and 60 mg/kg, respectively, administered by gavage for 28 days. The control group was given an equivalent volume of vehicle by gavage. All procedures were approved by the Animal Ethics Committee of Guizhou University of Traditional Chinese Medicine and adhered to the Guide for the Care and Use of Laboratory Animals (protocol code 20220158).

### 2.3. Assessment of Liver Injury Markers

Serum samples were analyzed to evaluate liver damage by measuring ALT and AST activities with a BS-240VET biochemical autoanalyzer (MAIRI, Shenzhen, China).

### 2.4. Histopathological Analysis of Liver Tissue

After the mice were sacrificed, liver tissues were promptly fixed in 10% formalin, embedded in paraffin, and sectioned. The sections were dewaxed, stained with hematoxylin and eosin, and visualized with a BA210 Digital Microsystem (Fuzhou, China).

### 2.5. Toxicokinetics Investigation

Blood samples from mice in the ME group were collected before dosing and at specific intervals (0.083, 0.17, 0.25, 0.5, 1.0, 2.0, 4.0, 6.0, 8.0, 10.0, 12.0, and 24.0 h) on Days 1 and 28. These samples were placed into heparinized Eppendorf tubes and immediately centrifuged at 12,000 rpm for 5 min.

For analysis, 100 µL of plasma and 20 µL of 0.2 μg/mL linarin (internal standard) were combined in a 96-well plate and shaken for 3 min. Protein precipitation was performed by adding 400 µL of acetonitrile, shaking for 10 min, centrifuging at 4000 rpm for 10 min, and then collecting the supernatant for UPLC-MS/MS analysis [26].

UPLC-MS/MS was conducted on an AB SCIEX system (USA) with an ESI ion source in positive ion mode. Separation was achieved using an Agilent ZORBAX-SB Column-C_18_ (2.1 × 50 mm, 3.5 μm) at a flow rate of 0.5 mL/min. The mobile phase comprised 5 mM ammonium acetate (A, 45%) and acetonitrile (B, 55%). ME and the internal standard were detected in multiple reaction monitoring mode.

### 2.6. Metabolomics Profiling

Liver tissues from the control and ME-H groups were subjected to metabolomics analysis. Post-sacrifice, the liver samples were collected, homogenized with five ceramic beads in 200 μL of water, and analyzed using UHPLC-Q-TOF MS. To ensure analytical consistency, 10 μL from each sample was pooled as a quality control.

Chromatographic separation utilized a 2.1 mm × 100 mm ACQUITY UPLC BEH Amide 1.7 µm column (Waters, Ireland). The mobile phase included 25 mM ammonium hydroxide and 25 mM ammonium acetate in water (A) and acetonitrile (B). The gradient started at 95% B for 0.5 min, then linearly reduced to 65% B over 6.5 min, dropped to 40% B in 1 min, held for 1 min, and returned to 95% B in 0.1 min. ESI source parameters were optimized as follows: Ion Source Gas2 (Gas2) and Ion Source Gas1 (Gas1) at 60, source temperature at 600 °C, IonSpray Voltage Floating (ISVF) at ±5500 V, and curtain gas (CUR) at 30. Mass spectrometry scanned the m/z range of 60-1000 Da with a TOF MS accumulation time of 0.20 s/spectrum [27].

### 2.7. 16S rRNA Gene Sequencing

Cecal contents from the control and ME-H groups were used for gut microbiota analysis. DNA was extracted with the Monarch DNA Gel Recovery Kit, and its concentration and purity were measured using a NanoDrop 2000 (Thermo Scientific, Waltham, MA, USA). PCR amplification was conducted with 15 µL of Phusion^®^ High-Fidelity PCR Master Mix (New England Biolabs, Ipswich, MA, USA). The resulting PCR products were purified, quantified, and converted into a sequencing library (SMRT Bell). Library preparation involved binding with the PacBio Binding Kit, and the final product was purified using AMpure PB Beads before sequencing on a Sequel II sequencer [28].

### 2.8. Statistical Analysis

Data analysis was performed using a *t*-test with GraphPad Prism (GraphPad 9.0 Software Inc., La Jolla, CA, USA), with significance set at *p* < 0.05. Following sum normalization, orthogonal partial least-squares discriminant analysis (OPLS-DA) was conducted. Spearman’s correlation tests were used to assess the relationship between the microbiome and liver metabolomics.

## 3. Results

### 3.1. Hepatotoxic Effects of ME in Mice

**Figure 1:**
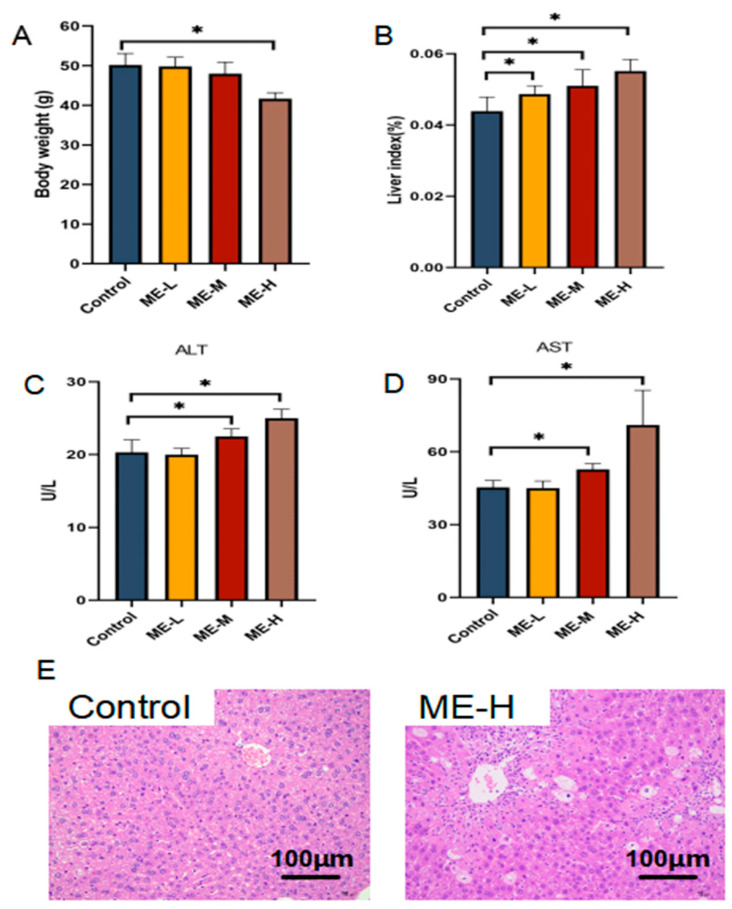
Hepatotoxicity induced by ME in mice after 28 days of administration. (**A**) Body weight. (**B**) Liver index. (**C**) Serum ALT levels. (**D**) Serum AST levels. (**E**) H&E staining of liver tissue. * *p* < 0.05 vs. control group.

Over 28 days of ME treatment, control mice maintained normal activity and health. In contrast, mice treated with oral ME, especially in the ME-H group, experienced a reduced appetite and a marked reduction in body weight compared to controls (Figure 1A). The liver index (liver weight/body weight × 100%) was significantly elevated in the ME-L, ME-M, and ME-H groups relative to controls (Figure 1B). Elevated ALT and AST levels were observed in the ME-M and ME-H groups compared to controls (Figure 1C,D). The ME-L group showed no significant deviations in body weight or biochemical parameters from controls. The histological analysis revealed normal hepatocyte morphology in the ME-L and ME-M groups. In contrast, the ME-H group exhibited pronounced vacuolation and hepatocellular steatosis after 28 days of high-dose ME treatment (60 mg/kg) (Figure 1E). These results demonstrate that ME administration at a high dose for 28 days induced considerable liver damage in mice.

### 3.2. Toxicokinetics Results

**Figure 2:**
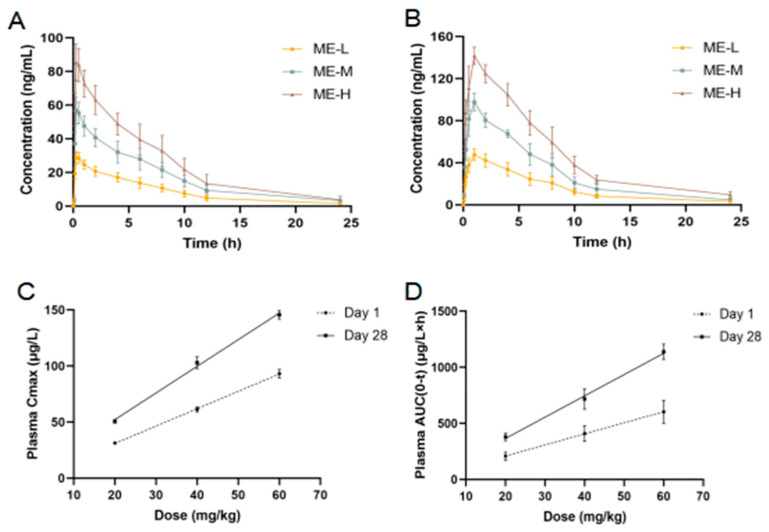
Toxicokinetics of ME in mice. (**A**) Plasma concentration–time curves of ME on Day 1 following oral administration (mean ± SD, *n* = 6). (**B**) Plasma concentration–time curves of ME on Day 28 following oral administration (mean ± SD, *n* = 6). (**C**) Peak plasma concentrations of ME at various doses. (**D**) Area under the plasma concentration–time curve (AUC) after oral ME administration.

Figure 2A,B depict the plasma concentration–time profiles of ME across different dosage groups. The administration of ME at 20, 40, and 60 mg/kg led to a plasma concentration pattern characterized by an initial rise followed by a decline on both Day 1 and Day 28. On both days, the peak plasma concentration (Cmax) and total exposure (AUC) increased with dosage levels (Figure 2C,D).

The toxicokinetic parameters for ME were analyzed using DAS 2.1.1 software. Tmax and Cmax were derived from observed data, and AUC0-t was calculated using the trapezoidal method (Table 1). On Day 28, Tmax showed a notable increase compared to Day 1. Both AUC and Cmax on Day 28 were significantly higher than on Day 1, with a dose-dependent manner. Peak plasma concentrations of ME occurred at 0.38 ± 0.14 h on Day 1, while on Day 28, peaks were observed between 0.92 and 1.08 h post-administration.

### 3.3. Metabolomics Analysis Results

**Figure 3:**
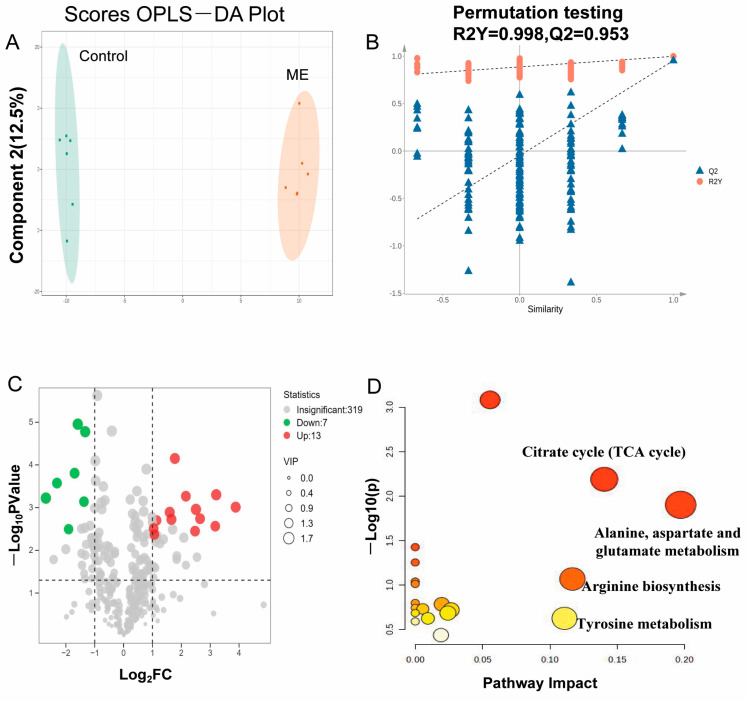
Impact of ME (60 mg/kg) on liver metabolites in mice. (**A**) OPLS−DA score plots of differential metabolites between ME and control groups. (**B**) Scatter plots from statistical validation using 200× permutation tests. (**C**) Volcano plot showing differential metabolites between ME and control groups. (**D**) Enrichment analysis of metabolic pathways affected by ME.

To identify metabolites linked to ME-induced hepatotoxicity, liver tissues from control and ME-treated (60 mg/kg) mice were analyzed using non-targeted metabolomics. Figure 3A shows that the OPLS-DA score plots distinctly separated the control from the ME group. A model validation via permutation tests confirmed the reliability, with R² values surpassing Q^2^ values and a Q^2^ regression line intercepting below zero, indicating no overfitting (Figure 3B).

The VIP (variable importance in the projection) values from the OPLS-DA models were used to assess variable contributions, and differential metabolites were visualized using a volcano plot (Figure 3C). Twenty metabolites were identified as biomarkers differentiating ME from control groups based on VIP ≥ 1.5, *p* < 0.05, and log_2_ (fold change) ≥ 1 or log_2_ (fold change) ≤ −1 (Table 2, Figure S1). Significantly upregulated metabolites included fatty acids and amino acids such as l-Glutamic acid, levodopa, d-alanyl-d-alanine, docosapentaenoic acid, and adrenic acid. To clarify the metabolic significance of these metabolites, a pathway analysis was conducted with MetaboAnalyst 6.0 (https://www.metaboanalyst.ca, accessed on 12 August 2024). Pathways with an impact value above 0.10 were selected (Figure 3D). This analysis indicated that ME administration affected the TCA (tricarboxylic acid) cycle, alanine–aspartate–glutamate metabolism, arginine biosynthesis, and tyrosine metabolism.

### 3.4. Impact of ME on Gut Microbiota

**Figure 4:**
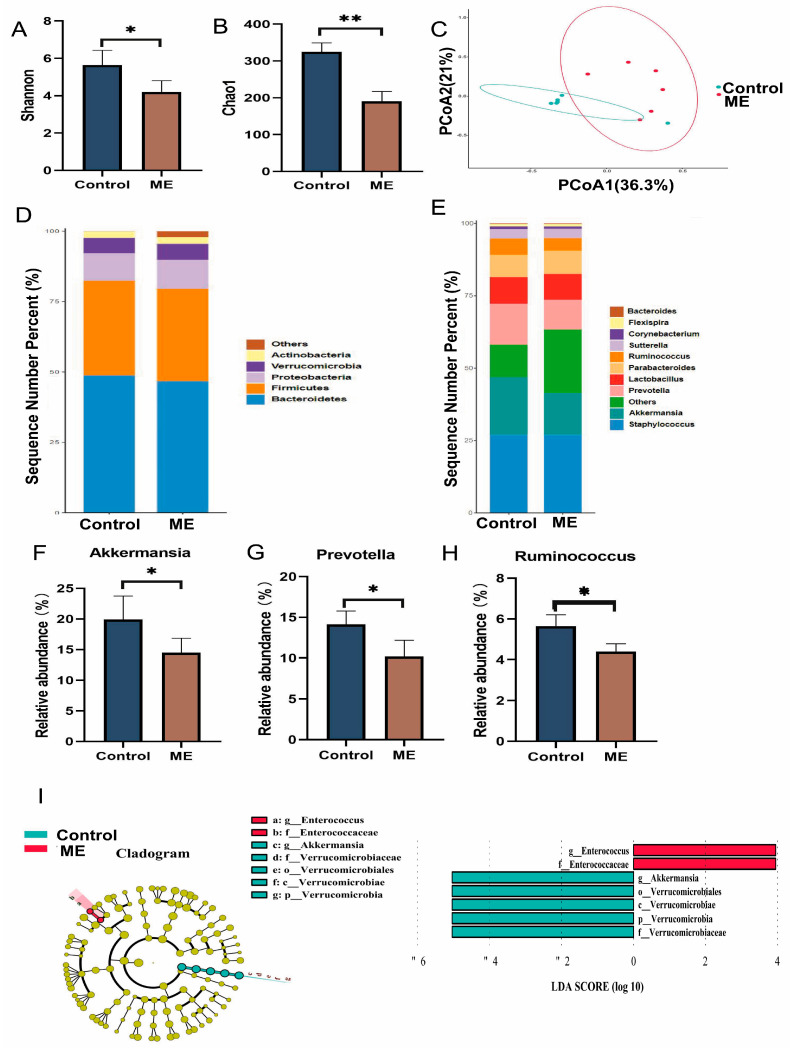
Effect of ME on gut microbiota composition in mice. (**A**) and (**B**) Shannon (diversity) and Chao 1 (richness) alpha diversity indices. (**C**) PCoA analysis based on Bray–Curtis dissimilarity. (**D**) Relative abundance of microbial taxa at the phylum level. (**E**) Relative abundance of microbial taxa at the genus level. (**F**) Abundance of Akkermansia. (**G**) Abundance of Prevotella. (**H**) Abundance of Ruminococcus. (**I**) Differentially abundant microbial taxa and multilevel species bar chart from LEfSe analysis, showing genus−level biomarkers with an LDA score > 2. * *p* < 0.05 vs. control group. ** *p* < 0.01 vs. control group.

The 16S rRNA genes of intestinal microbiota in control and ME-treated (60 mg/kg) mice were analyzed through high-throughput sequencing. The ME group showed markedly lower Shannon and Chao1 indices compared to controls (Figure 4A,B). The principal coordinates analysis (PCoA) with Bray–Curtis distances indicated clear differences in the microbiota composition between the two groups, reflecting significant microbiota shifts due to ME treatment (Figure 4C). Although ME treatment reduced the relative abundances of Bacteroidetes and Firmicutes at the phylum level, these changes were not statistically significant (Figure 4D). At the genus level, however, Akkermansia, Prevotella, and Ruminococcus significantly decreased in the ME group (Figure 4E–H). The linear discriminant analysis (LDA) effect size (LEfSe) revealed Akkermansia as a key genus in the control group, while Enterococcus was more prevalent in the ME group (Figure 4I).

**Figure 5:**
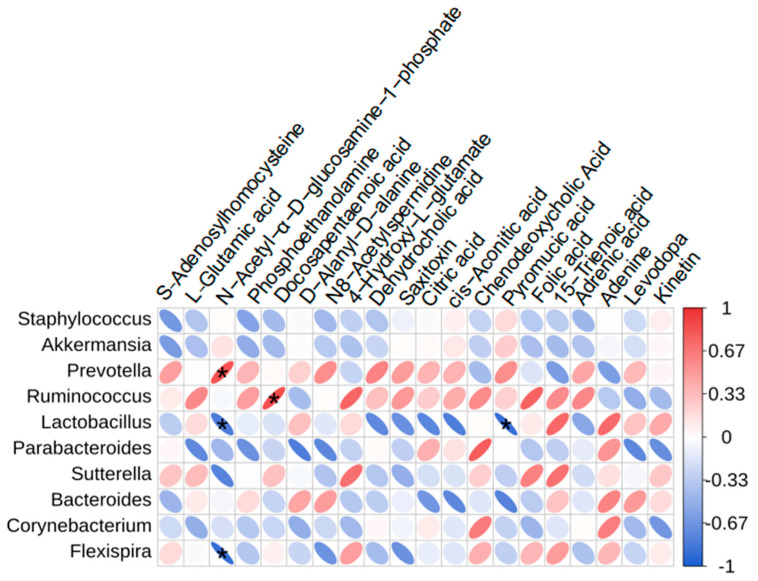
Correlations between gut microbiota abundance at the genus level and liver metabolites. * *p* < 0.05.

Spearman’s correlation analysis explored the relationship between changes in relative abundance of gut microbiota and relative content of liver metabolites (Figure 5). This analysis suggested that specific gut bacteria, such as Prevotella and Lactobacillus, correlate with liver metabolites during ME treatment. Specifically, Prevotella showed a positive correlation with N-acetyl-α-D-glucosamine 1-phosphate, while Lactobacillus negatively correlated with both N-acetyl-α-D-glucosamine 1-phosphate and pyromucic acid, indicating their involvement in ME-induced hepatotoxicity.

## 4. Discussion

The mechanism behind ME-induced liver injury, although commonly observed, is not fully understood. Our study revealed that administering ME (60 mg/kg) orally resulted in substantial increases in the liver index, ALT, and AST levels. After 28 days of treatment, mice showed significant liver damage characterized by vacuolation and hepatocellular steatosis.

We combined toxicokinetics, metabolomics, and gut microbiota analyses to investigate the hepatotoxic mechanisms of ME from different perspectives. The plasma concentrations of ME increased with dosage (20, 40, and 60 mg/kg) on both Day 1 and Day 28. ME may induce the activity of CYP3A4 enzymes in mice. Due to the accelerated metabolism, the speed of metabolism and clearance of ME after entering the blood circulation becomes faster, which slowed down the rate of increase in plasma concentration of ME, thus leading to a delay in Tmax [29,30]. Elevated Tmax, AUC, and Cmax on Day 28 compared to Day 1 indicate dose-dependent increases and delayed Tmax, suggesting that prolonged exposure and higher plasma accumulation may enhance side effects and contribute to liver toxicity [31,32].

The metabolomic analysis identified significant disruptions in key metabolic pathways due to ME, including the TCA cycle; alanine, aspartate, and glutamate metabolism; arginine biosynthesis; and tyrosine metabolism, with 20 potential biomarkers highlighted. These disruptions are central to ME-induced hepatotoxicity, leading to structural abnormalities in liver cells [33,34]. Prior research has shown that mitochondrial enzyme activity related to the TCA cycle is impaired in liver injury, affecting energy metabolism [35,36,37,38]. Our findings further demonstrate that ME administration alters amino acid metabolism pathways, specifically those involving alanine, aspartate, glutamate, arginine, and tyrosine, contributing to liver injury.

The contribution of gut microbiota to hepatotoxicity is not yet fully understood. Our study revealed that a 28-day oral administration of ME notably decreased α-diversity and altered β-diversity in the gut microbiota. Specifically, ME treatment led to significant reductions in Akkermansia, Prevotella, and Ruminococcus. This decline in Akkermansia was further validated by the LEfSe of relative abundance data. Akkermansia, which depends on the intestinal mucous layer, is crucial for gut health [39]. Its reduction may compromise the intestinal barrier, potentially causing inflammation and related disorders [40,41,42]. Additionally, mice experienced severe diarrhea and weight loss, suggesting that prolonged ME administration might lead to hepatotoxicity through the gut–liver axis.

Correlation analysis identified significant links between Prevotella and N-acetyl-α-D-glucosamine 1-phosphate, and between Lactobacillus and both N-acetyl-α-D-glucosamine 1-phosphate and pyromucic acid, indicating their possible roles in hepatotoxicity. ME may cause liver damage by disrupting the balance of Akkermansia, Prevotella, and Ruminococcus and by affecting amino acid and TCA cycle metabolism. Despite these findings, this study is limited to correlating ME, gut microbiota, and liver metabolites. Future research should utilize pseudo-germ-free model mice to explore the relationships between the microbiome, metabolic changes, and hepatotoxicity [43].

## 5. Conclusions

This study utilized a multifaceted approach, incorporating toxicokinetics, metabolomics, and gut microbiota analysis, to elucidate the mechanism of ME-induced hepatotoxicity in mice. The significant elevation in ALT and AST levels, coupled with notable weight loss, confirmed severe liver damage caused by ME. Repeated ME administration resulted in prolonged Tmax and increased plasma levels. ME-induced toxicity was associated with changes in Akkermansia, Prevotella, and Ruminococcus abundance, and disruptions in amino acid and TCA cycle metabolism. Further research is needed to clarify the relationship between gut microbiota and metabolic alterations. Ensuring gut microbiota stability during ME exposure is crucial for understanding its hepatotoxic effects.

## Figures and Tables

**Table 1:** Toxicokinetic profile of ME across all doses in mice plasma. (*n* = 6, mean ± SD).

Time	Dose (mg/kg)	T1/2 (h)	Tmax (h)	Cmax (μg/L)	AUC _(0–∞)_	AUC _(0–t)_ (μg/L·h)
Day 1	20	5.56 ± 1.25	0.375 ± 0.137	31.258 ± 1.249	224.215 ± 41.809	207.833 ± 35.945
	40	6.02 ± 0.74	0.375 ± 0.137	61.313 ± 2.067	444.941 ± 83.842	408.773 ± 69.847
	60	4.96 ± 1.98	0.375 ± 0.137	93.237 ± 3.791	641.660 ± 125.788	602.684 ± 103.124
Day 28	20	5.53 ± 1.84	1.083 ± 0.492 *	50.602 ± 1.557 *	401.429 ± 36.973	379.291 ± 33.738 *
	40	5.38 ± 1.55	0.917 ± 0.204 *	102.89 ± 5.241 *	756.641 ± 108.84	717.844 ± 90.372 *
	60	4.95 ± 1.68	0.917 ± 0.204 *	145.7 ± 3.920 *	1193.716 ± 111.91	1138.405 ± 69.389 *

* *p* < 0.05, compared to the same dose group on Day 1 after administration of ME.

**Table 2:** Identified potential biomarkers in liver.

No.	Metabolite	m/z	RT (min)	VIP	Fold Change	Trend
1	S-Adenosylhomocysteine	193.06841	2.321	1.697	0.398	↓ *
2	Adenine	136.06219	4.807	1.722	0.333	↓ *
3	Dehydrocholic acid	403.24514	6.223	1.527	0.267	↓ *
4	Saxitoxin	282.13191	5.616	1.574	0.386	↓ *
5	Folic acid	440.13283	5.075	1.615	0.202	↓ *
6	Chenodeoxycholic Acid	437.29076	7.700	1.718	0.155	↓ *
7	4-Hydroxy-L-glutamate	162.04129	2.371	1.620	0.308	↓ *
8	L-Glutamic acid	148.06047	1.299	1.502	2.216	↑ *
9	Phosphoethanolamine	142.02644	1.287	1.588	4.464	↑ *
10	Levodopa	215.10301	2.159	1.562	9.043	↑ *
11	D-Alanyl-D-alanine	161.09236	2.150	1.634	14.759	↑ *
12	N8-Acetylspermidine	188.17607	1.251	1.594	6.275	↑ *
13	Kinetin	216.08688	2.276	1.632	3.427	↑ *
14	Citric acid	191.01896	1.802	1.685	9.249	↑ *
15	cis-Aconitic acid	173.00828	2.149	1.640	3.153	↑ *
16	Pyromucic acid	111.00764	2.024	1.578	5.566	↑ *
17	N-Acetyl-α-D-glucosamine-1-phosphate	300.04853	1.303	1.576	2.058	↑ *
18	15-Trienoic acid	293.21237	7.342	1.538	2.098	↑ *
19	Docosapentaenoic acid	329.24852	9.892	1.638	3.029	↑ *
20	Adrenic acid	331.26399	10.390	1.687	5.686	↑ *

* *p* < 0.05, vs. control group. ↑ Represents an increase and ↓ represents an decrease for the comparison of ME group vs. control group. RT: retention time. VIP: variable importance in the projection.

## Data Availability

The results are available on reasonable request from the last author of this study.

## Supplementary Materials

The following supporting information can be downloaded at https://www.mdpi.com/article/10.3390/cimb46100673/s1, Figure S1: identification of 20 potential biomarkers distinguishing ME and control groups.
