# Supplementary figures and images for: Hepatotoxicity Induced by Methyl Eugenol: Insights from Toxicokinetics, Metabolomics, and Gut Microbiota
Source: Curr Issues Mol Biol. 2024 Oct 11;46(10):11314–25. doi: 10.3390/cimb46100673 (PMC11506582; doi:10.3390/cimb46100673)

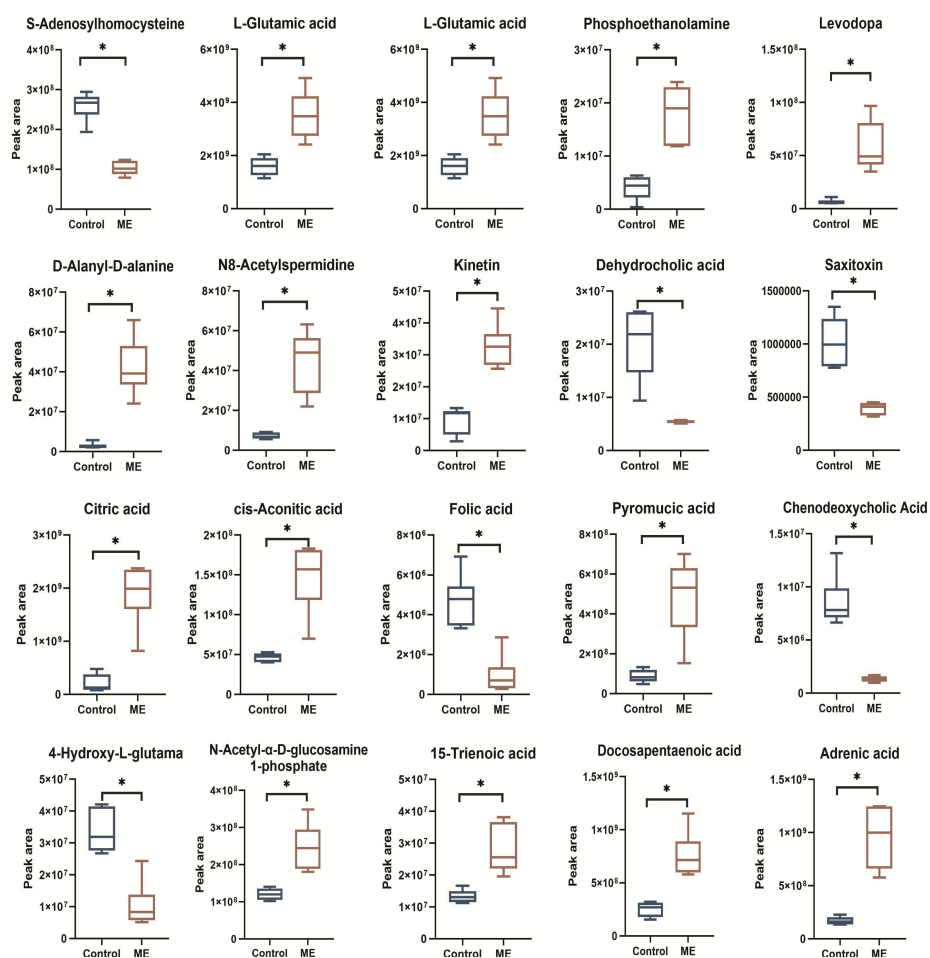

**Figure S1.** Identification of 20 potential biomarkers distinguishing ME and control groups.

Supplement: Supplementary file 1 [file cimb-46-00673-s001.zip › cimb-3243968-supplementary.pdf]
